# British Home and Hospital for Incurables, Streatham

**Published:** 1918-06-08

**Authors:** 


### THE EDITOR'S LETTER-BOX.

### British Home and Hospital for Incurables, Streatham.

*To the Editor of* THE HOSPITAL.

SIR,—I should like to be allowed to bring before the notice of your readers the urgent need of the British Home and Hospital for Incurables, Streatham, for help. For the year ending March 31, 1918, there was a deficit of £5,814, and it is the earnest desire of the committee and myself that this deficit should be met at once, and we are therefore making a great effort to raise the sum of £6,000 for this purpose, and I plead for your readers' kind and generous support.

In addition to the usual work of the institution, upwards of 1,000 wounded soldiers have received treatment at the Home at Streatham.

This appeal has the approval of Her Majesty Queen Alexandra, who has done me the honour graciously to send me a letter in its support, a copy of which I enclose, and I shall be much obliged if you will kindly publish it with this appeal.—Yours faithfully,

May 29, 1918. PORTLAND, President.

